# Educational Artificial Intelligence Software to Support Assessment of Atopic Dermatitis Severity

**DOI:** 10.1111/1346-8138.70434

**Published:** 2026-07-30

**Authors:** Kenichi Yamanaka, Yağmur Pak, Yoshihiro Inda, Toshiaki Nomi, Keisuke Ogaki, Masato Hoshi, Kouki Nakamura

**Affiliations:** ^1^ Specialty Care Medical Affairs Pfizer Japan Inc. Tokyo Japan; ^2^ Medical Enablement & Quality, Chief Medical Office Pfizer Belgium Brussels Belgium; ^3^ Digital Pfizer Japan Inc. Tokyo Japan; ^4^ M3 Inc. Tokyo Japan

**Keywords:** artificial intelligence, atopic dermatitis, convolutional neural networks, medical education, severity of illness index

## Abstract

Accurate assessment of the severity of atopic dermatitis is crucial for guiding treatment. However, the Eczema Area and Severity Index (EASI), a widely used clinical assessment tool, relies on subjective visual scoring, which can lead to inter‐rater variability. This study aimed to evaluate the performance of convolutional neural network‐based artificial intelligence software in assessing atopic dermatitis severity from uncropped, unmasked skin images acquired under uncontrolled conditions. In this prospective, multicenter observational study involving 16 Japanese institutions, 1016 patients with atopic dermatitis provided 3676 smartphone‐captured skin images. A ConvNeXt‐based artificial intelligence model was trained to predict the severity scores for four established signs of the EASI—erythema, papulation/edema, excoriation, and lichenification—and these were evaluated using receiver operating characteristic analysis against dermatologist assessments. For an EASI component score of ≥ 2, the area under the receiver operating characteristic curve ranged from 0.825 to 0.860 across signs, with sensitivities of 0.745–0.873 and specificities of 0.675–0.764. For a component severity score of 3, the area under the receiver operating characteristic curve exceeded 0.900, with sensitivity up to 1.000 and specificity up to 0.906. The area under the receiver operating characteristic curve for a severity score of ≥ 1 remained consistently > 0.700. Among the four evaluated signs, erythema demonstrated the highest accuracy, whereas papulation/edema showed the lowest. This ConvNeXt‐based artificial intelligence software demonstrated robust performance in evaluating atopic dermatitis severity using real‐world images. As an educational tool, it may support standardized training and reduce inter‐rater variability in EASI sign scoring. It is not intended for direct clinical decision‐making at this stage.

## 1 Introduction

Atopic dermatitis (AD) is a common chronic inflammatory skin condition characterized by intensely pruritic, eczematous lesions that follow a relapsing–remitting course [1]. Its clinical manifestations range from mild, localized involvement to widespread, treatment‐resistant disease. Moderate‐to‐severe AD poses a considerable burden on patients, often disrupting sleep, impairing daily activities, and significantly reducing their quality of life [2]. Accurate assessment of disease severity is essential for guiding treatment decisions and monitoring therapeutic outcomes.

The Eczema Area and Severity Index (EASI) is a validated, semi‐quantitative scoring system widely used to evaluate AD severity [3, 4]. It assesses four signs—erythema, papulation/edema, excoriation, and lichenification—across four body regions to generate a composite score. The Harmonizing Outcome Measures for Eczema initiative recommends the EASI as the core outcome measure for assessing AD severity in both clinical trials and practice [5]. However, EASI scoring relies on visual estimation by clinicians, which introduces subjectivity and potential inter‐rater variability [3, 6, 7]. This limitation highlights the need for standardized training and objective evaluation methods to ensure consistency in EASI assessments.

Recent advancements in artificial intelligence (AI), particularly convolutional neural networks (CNNs), have demonstrated dermatologist‐level performance in the diagnosis of various dermatologic conditions. CNN‐based models have shown high accuracy in detecting skin cancer [8], onychomycosis [9], and psoriasis [10]. Furthermore, CNN‐based models trained on large clinical image datasets have achieved performance comparable to expert assessments. Moreover, CNN‐assisted training has been shown to enhance diagnostic accuracy and consistency among non‐specialists, such as general practitioners [11]. Building on these developments, CNN‐based tools may offer a promising approach to standardizing EASI scoring by improving inter‐rater reliability and supporting clinical education.

Although AI‐based methods have shown promising diagnostic accuracy in evaluating EASI severity, previous studies have relied on cropped images of AD lesions or expert‐generated pixel‐level segmentation masks [12, 13, 14], which limited scalability. In contrast, we hypothesized that an AI software capable of training and inference directly from uncropped, unmasked smartphone images acquired under variable lighting and viewing angles could enable a low‐cost, highly adaptable approach scalable to diverse real‐world clinical settings and large‐scale multicenter studies.

In this study, we developed a supervised learning model that ingests uncropped, unmasked smartphone‐captured skin images as inputs; uses as training labels the consensus severity grades (0–3) for the four EASI signs (erythema, papulation/edema, excoriation, and lichenification) provided by a Central Assessment Committee of board‐certified dermatologists; and outputs a continuous severity score for each sign. The network was therefore trained as a regression model to approximate expert severity grading, rather than to perform diagnostic classification of AD.

The objective of this multicenter observational study was to assess the performance of AI software in evaluating the severity of four clinical features of AD—erythema, papulation/edema, excoriation, and lichenification—as defined by the EASI scoring system.

## 2 Methods

### 2.1 Study Design

This was a prospective, cross‐sectional, non‐interventional, multicenter observational study conducted across 16 institutions in Japan. Patients were enrolled between October 2023 and March 2024. After obtaining written informed consent and confirming eligibility, skin images and data on patient demographics, and imaging‐related metadata were collected. The consent and data acquisition process were completed on the same day.

The primary endpoint was the accuracy of the AI software in identifying AD signs with an EASI component score of ≥ 2. Secondary endpoints included accuracy for detecting severity scores of 3 and ≥ 1. All AI‐predicted severity scores were compared with reference evaluations established through consensus by a Central Assessment Committee composed of board‐certified dermatologists, helping to mitigate potential inter‐rater variability and subjective bias associated with EASI scoring.

### 2.2 Ethical Consideration

The study was conducted in accordance with the Declaration of Helsinki and international ethical guidelines, including those of the Council for International Organizations of Medical Sciences. It also adhered to the Ethical Guidelines for Medical and Biological Research Involving Human Subjects, along with relevant Japanese laws and regulations, including the Act on the Protection of Personal Information. All patients provided written informed consent, and the study protocol was approved by the Takahashi Clinic Ethical Committee. This study is reported in accordance with the Strengthening the Reporting of Observational Studies in Epidemiology statement.

### 2.3 Participants

To ensure feasibility, the target sample size was set at 1000 patients. Inclusion criteria were as follows: age ≥ 18 years at the time of consent, a current or past diagnosis of AD by a dermatologist, and provision of written informed consent prior to participation. AD was diagnosed by board‐certified dermatologists at each participating site as part of routine clinical practice. No single standardized set of diagnostic criteria was mandated by the study protocol. Exclusion criteria included the presence of concurrent skin conditions that could interfere with the assessment of AD severity via imaging, an evident EASI score of 0, and individuals directly involved in the conduct of the study, including their family members. Eligible patients treated at the 16 participating institutions and who provided written informed consent were sequentially enrolled during the study period through convenience sampling.

### 2.4 Skin Image Assessment

Skin images were captured using an iPhone SE2 (Apple Inc., Cupertino, CA, USA), with a maximum of four skin images per patient. Images were acquired by principal investigators or sub‐investigators from the head and neck, trunk, upper limbs, and lower limbs, as defined by the EASI scoring system. Within these anatomical regions, physicians selected representative lesion areas for imaging based on clinical judgment to best reflect disease severity. Scalp lesions were not collected; facial lesions were photographed when present. Investigators were instructed to capture images in which the target lesion was clearly visible, even if body hair was incidentally present, and to avoid including the eyes. If the eyes were captured, the eye region was masked during quality control. To ensure consistency in image quality and acquisition conditions, the smartphone was held approximately 10 cm from the skin surface during imaging, and a color calibration scale was included in all images for reference (Figure 1).

**FIGURE 1:**
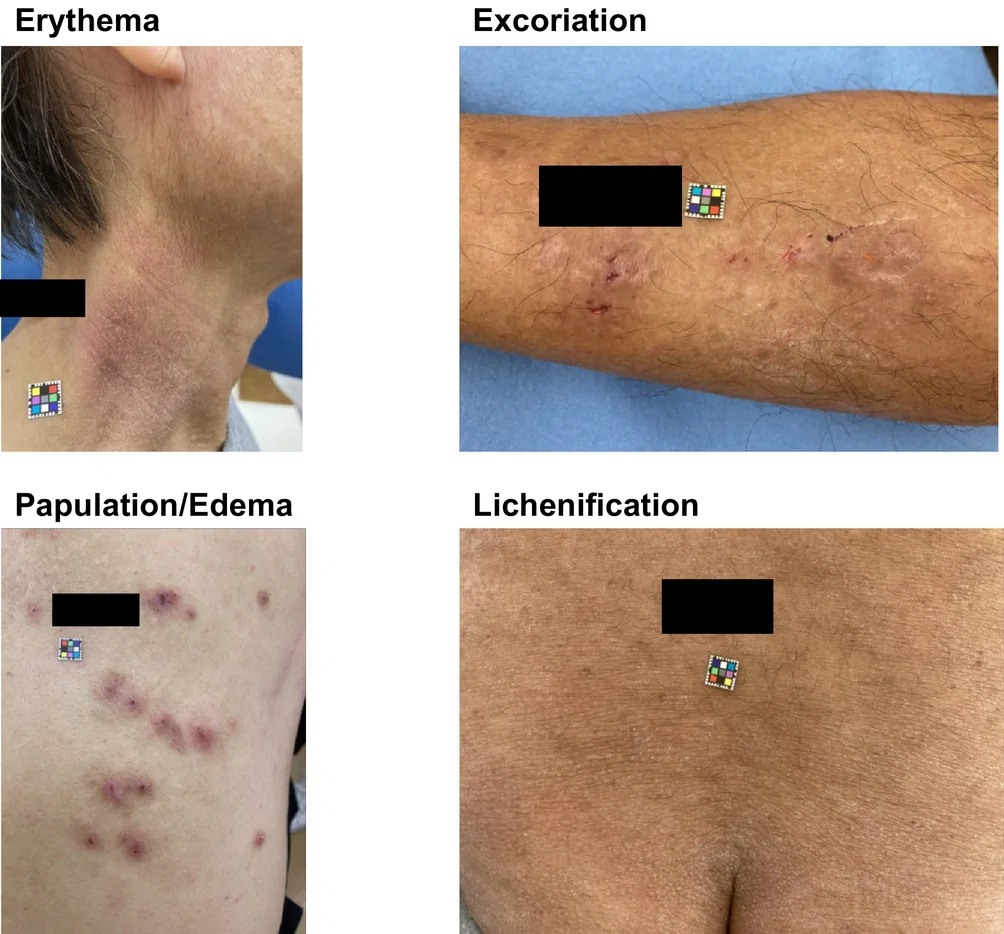
Representative clinical images of atopic dermatitis acquired in this study. These images are representative examples of the four Eczema Area and Severity Index signs: erythema, papulation/edema, excoriation, and lichenification. The images shown are cases in which the artificial intelligence software's assessment and the Central Assessment Committee's reference evaluation concurred on the Eczema Area and Severity Index component score of ≥ 2, the primary endpoint of this study. All images were captured on smartphones under variable lighting and angles, and were used for training, validation, and testing without cropping or masking.

A CNN‐based algorithm using the ConvNeXt architecture was employed to assess the severity of dermatological lesions in the images. ConvNeXt, a high‐performing ConvNet, rivals vision transformers in classification and segmentation tasks while retaining the efficiency and structural simplicity of conventional CNNs. Unlike transformers, ConvNeXt does not require mechanisms such as shifted window attention or relative position bias, making it suitable for scalable deployment [15]. The model was designed to simultaneously output severity scores for the four EASI signs: erythema, papulation/edema, excoriation, and lichenification (Figure 2).

**FIGURE 2:**
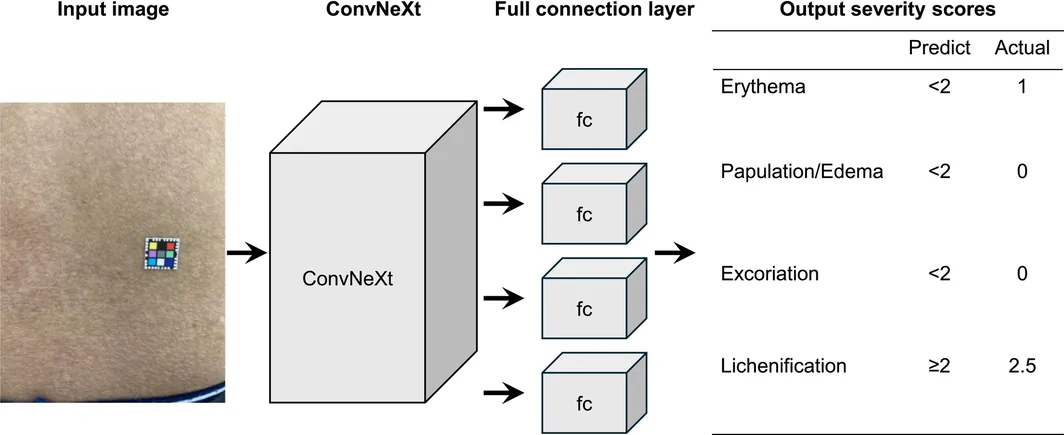
Artificial intelligence model architecture. The artificial intelligence model analyzes the input skin image using ConvNeXt to extract visual features. ConvNeXt offers high performance in classification and segmentation tasks, with structural simplicity and scalability compared to vision transformers. Following feature extraction, the model branches into four separate paths, each containing a fully connected layer (labeled as “fc”). These layers simultaneously calculate and output the severity scores for the four EASI signs: Erythema, papulation/edema, excoriation, and lichenification.

Each image was resized to 224 × 224 pixels and augmented through techniques such as random affine transformations, horizontal and vertical flipping, motion blur, Gaussian and median blur, histogram equalization, and optical/grid distortions. The model was trained to minimize mean squared error between predicted and reference scores across all sign domains. Training utilized the Adam optimizer with a learning rate of 0.0001 and was conducted on an NVIDIA Tesla T4 GPU (NVIDIA Corporation, Santa Clara, CA, USA). The dataset was split into training, validation, and test subsets, with five‐fold cross‐validation employed for robust performance evaluation (Figure 3). Test‐time augmentation was also applied: four versions of each test image (original, horizontally flipped, vertically flipped, and both) were processed, and the final prediction was derived by averaging the outputs.

**FIGURE 3:**
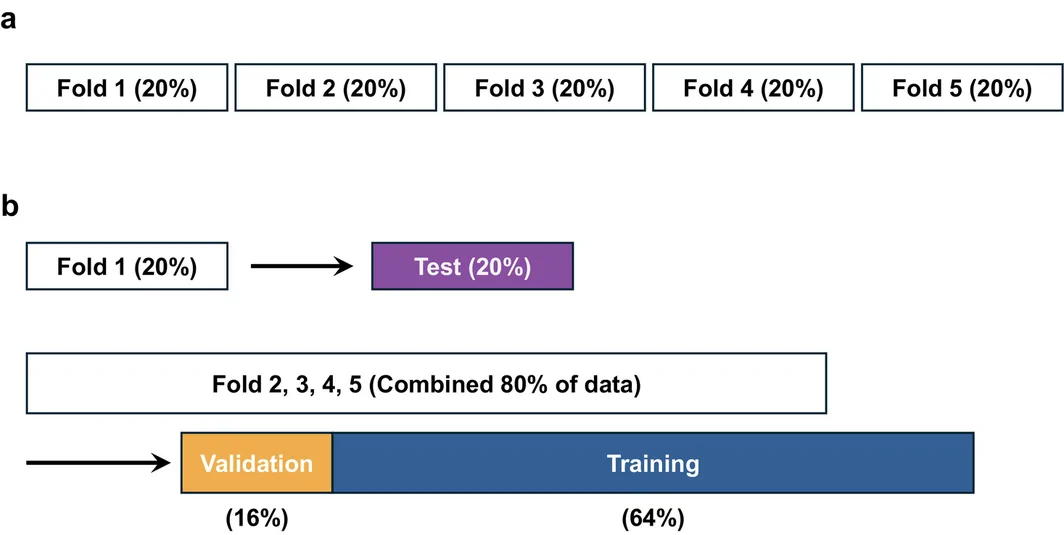
Five‐fold cross‐validation scheme used for model training and evaluation. (a) The entire dataset, stratified by patient, was partitioned into five equal folds, with each fold comprising 20% of the data. (b) In each of the five cross‐validation iterations, a single fold (20% of the data) was held out as the test set. The remaining four folds (80% of the data) were then combined and re‐split into a validation set (16% of the total data) and a training set (64% of the total data). This process was repeated until every fold served as the test set once.

### 2.5 Performance Evaluation of AI‐Based AD Severity Assessment

While the model outputs continuous values for each EASI sign using regression model, we additionally assessed clinically interpretable classification performance by thresholding these predictions at EASI‐relevant cut points (≥ 1, ≥ 2, and 3) and computing receiver operating characteristic (ROC)‐based metrics. This links the training objective with clinical decision thresholds used by raters.

ROC curves were generated by comparing AI‐predicted severity scores for each sign with the reference scores provided by the Central Assessment Committee. The area under the ROC curve (AUC), sensitivity, specificity, positive predictive value (PPV), and negative predictive value were calculated across 48 combinations, derived from four anatomical regions (head and neck, trunk, upper limbs, and lower limbs), four EASI signs (erythema, papulation/edema, excoriation, and lichenification), and three severity levels (≥ 1, ≥ 2, and 3). To establish the cutoff values for sensitivity and specificity, the Youden index was applied uniformly to each combination of region, sign, and severity level.

For each predicted severity score, the mean and standard deviation of the AUC, sensitivity, and specificity across the four body regions were calculated. The AUC values were interpreted using established criteria: 0.500–0.700, 0.700–0.900, and 0.900–1.000, indicating low, moderate, and high accuracy, respectively [16].

### 2.6 Statistical Analysis

Descriptive statistics were used to summarize patient characteristics. For a single missing age data point, descriptive statistics were calculated based on the available data. For continuous variables, measures such as mean, median, standard deviation, minimum, maximum, and interquartile range were reported, as appropriate. All statistical analyses were performed using SAS software (version 9.4; SAS Institute Inc., Cary, NC, USA).

## 3 Results

### 3.1 Patient Characteristics and Image Data

A total of 1018 patients were recruited. Of these, two were excluded due to protocol deviations, resulting in 1016 (662 males and 354 females) being included in the final analysis. The mean ± standard deviation age was 38.6 ± 13.6 years. Patient demographics and baseline characteristics are presented in Table 1.

**TABLE 1:** Patient background data.

Total number of patients	*n*	1016
Age, years old	*n*	1015
	Mean ± SD	38.6 ± 13.6
	Median	38.0
	Q1, Q3	27.0, 48.0
	Minimum, Maximum	18.0, 83.0
Sex	Male	662 (65.2)
	Female	354 (34.8)
History of AD, months	*n*	1016
	Mean ± SD	299.8 ± 189.3
	Median	288.0
	Q1, Q3	156.0, 444.0
	Minimum, Maximum	0.0, 876.0
Treatment for AD	No	12 (1.2)
	Yes	1004 (98.8)

*Note:* The Sex and Treatment for AD indicate the number (%) of patients. Regarding age, since data for one patient could not be obtained, the mean, median, Q1, Q3, minimum, and maximum were calculated from the data of 1015 patients. The history of AD, recorded in months, was based on routine‐care information available at the time of enrollment. A value of 0.0 months indicates < 1 month, such as in newly presenting patients diagnosed within the same month.

Abbreviations: AD, atopic dermatitis; Q1, first quartile; Q3, third quartile; SD, standard deviation.

From the enrolled cohort, 3681 skin images were collected. After excluding five images due to violations of inclusion criteria or conflict with exclusion criteria, 3676 images were retained for model training and evaluation (Figure 4).

**FIGURE 4:**
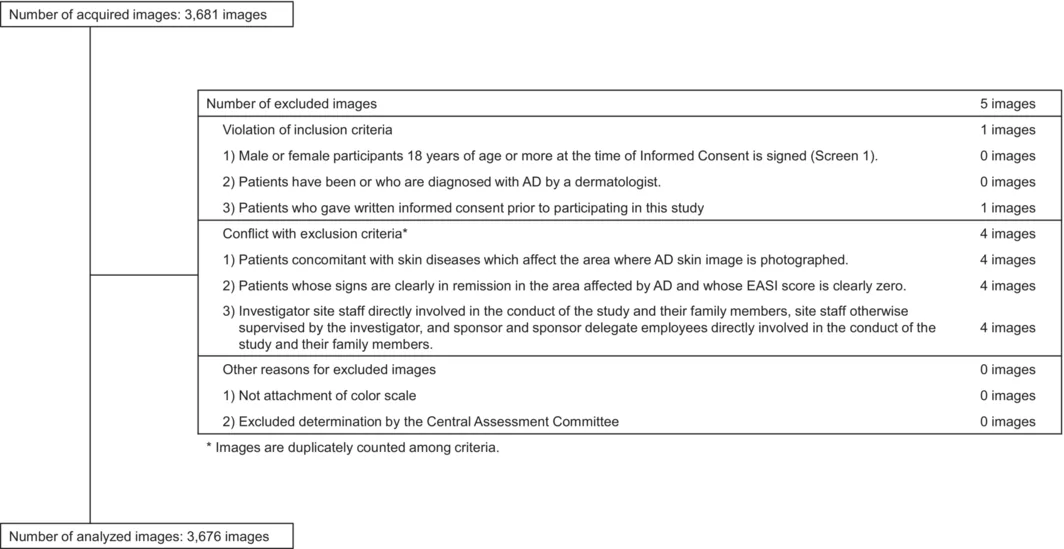
Flow chart of skin image processing and selection. A total of 3681 skin images were collected from patients with atopic dermatitis. Five images were excluded due to violations of inclusion criteria or conflicts with exclusion criteria. These included issues such as lack of consent and comorbid skin conditions. Some images met more than one exclusion criterion. The final dataset comprised 3676 images evaluated by the artificial intelligence model. AD, atopic dermatitis; EASI, Eczema Area and Severity Index.

The number of images and corresponding patients by anatomical region is summarized in Table 2. The distribution of image counts across severity levels for each of the four EASI signs is detailed in Table S1.

**TABLE 2:** Number of pictures and patients used for analysis.

	Number of pictures	Number of patients
Total	3676 (5)	1016 (2)
Head and neck	712 (1)	503 (0)
Trunk	1150 (1)	663 (0)
Upper limb	1244 (0)	747 (0)
Lower limb	570 (3)	402 (2)

*Note:* The values are given as the number of pictures or patients (excluded pictures or patients). Since multiple regions were imaged for the same patient, the total for each region and the total number of patients differ. Additionally, due to deviations from the data collection procedure, images could not be collected from 10 patients out of 1016.

### 3.2 Performance of the AI Software for Detecting Signs With an EASI Component Score of ≥ 2

The ROC curves for the four representative signs—erythema, papulation/edema, excoriation, and lichenification—are shown in Figure 5. The performance of the AI software in detecting signs with an EASI component score of ≥ 2 was evaluated across all four signs. The AUC values ranged from 0.825 to 0.860, with corresponding sensitivities and specificities ranging from 0.745 to 0.873 and 0.675 to 0.764, respectively. Among the signs, erythema demonstrated the highest accuracy (AUC = 0.860), whereas papulation/edema showed the lowest (AUC = 0.825). The detailed results are provided in Table 3. A detailed comparison between the AI‐predicted severity and the reference assessments made by the Central Assessment Committee is presented in Tables S2–S5 for each sign.

**FIGURE 5:**
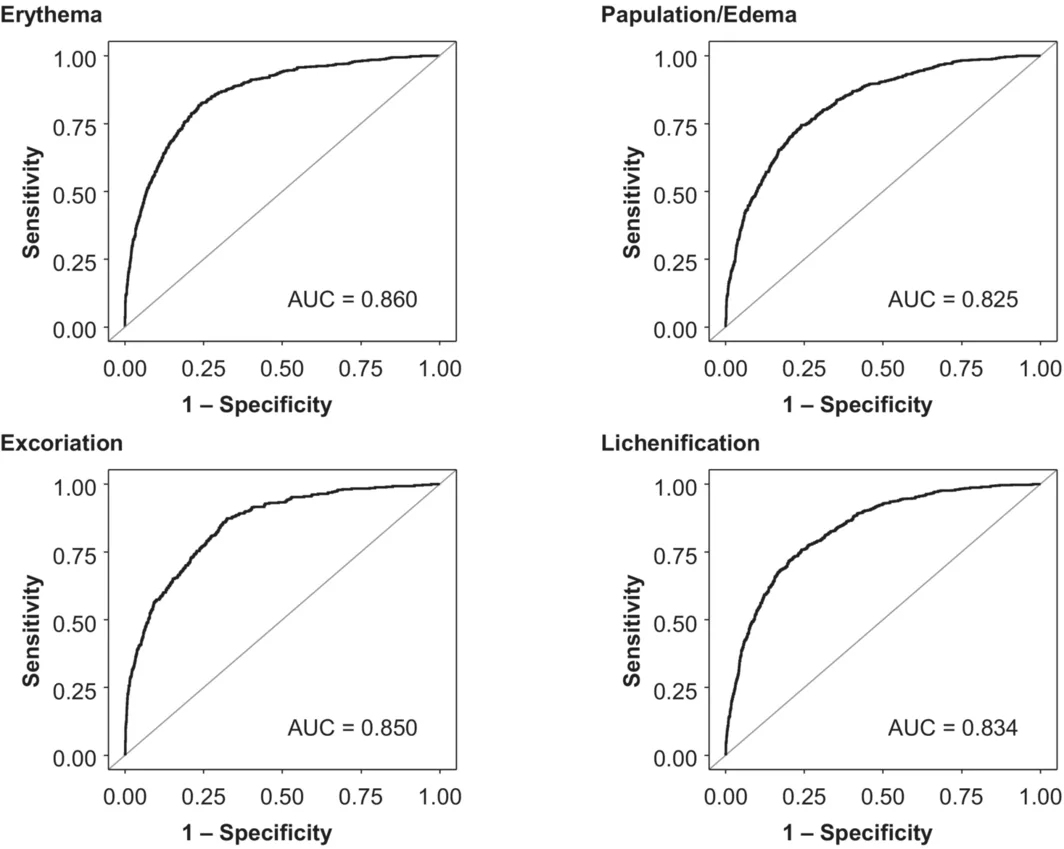
Receiver operating characteristic curves for artificial intelligence‐based assessment of an EASI component score of ≥ 2. Each curve represents the ability of the model to identify one of the four clinical signs (erythema, papulation/edema, excoriation, and lichenification). The vertical axis indicates sensitivity, and the horizontal axis shows one minus specificity. The area under the receiver operating characteristic curve (AUC) is 0.860 for erythema, 0.825 for papulation/edema, 0.850 for excoriation, and 0.834 for lichenification. AUC, area under the receiver operating characteristic curve; EASI, Eczema Area and Severity Index.

**TABLE 3:** Diagnostic accuracy of AI software for each sign of atopic dermatitis with an EASI component score of ≥ 2.

	AUC	95% CI		Sensitivity	Specificity	PPV	NPV
		Lower	Upper				
Erythema	0.860	0.845	0.875	0.818	0.764	0.444	0.948
Papulation/Edema	0.825	0.809	0.840	0.745	0.759	0.497	0.903
Excoriation	0.850	0.831	0.869	0.873	0.675	0.257	0.977
Lichenification	0.834	0.819	0.849	0.758	0.759	0.460	0.920

Abbreviations: AI, artificial intelligence; AUC, area under the receiver operating characteristic curve; CI, confidence interval; EASI, Eczema Area and Severity Index; NPV, negative predictive value; PPV, positive predictive value.

### 3.3 Performance of the AI Software for Detecting Signs With an EASI Component Score of 3

For detecting signs with an EASI component score of 3, the AI software demonstrated AUCs above 0.900 across all four signs. The AUC values ranged from 0.901 to 0.959, with excoriation showing the highest accuracy and papulation/edema showing the lowest. Notably, the model achieved a sensitivity of 1.000 for excoriation and maximum specificity of 0.906 for erythema. The detailed results are presented in Table S6.

### 3.4 Performance of the AI Software for Detecting Signs With an EASI Component Score of ≥ 1

The performance of the AI software in identifying signs with an EASI component score of ≥ 1 was assessed. The AUC values ranged from 0.778 to 0.832. In contrast to the other severity levels, papulation/edema demonstrated the highest accuracy for this endpoint, whereas lichenification showed the lowest. The corresponding sensitivities and specificities ranged from 0.692 to 0.713 and 0.718 to 0.803, respectively. The detailed results are presented in Table S7.

## 4 Discussion

In this study, we developed an AI software to assess the severity of four signs, including erythema, papulation/edema, excoriation, and lichenification, in AD and evaluated its performance across varying severity levels. Although the EASI score is widely used to assess AD severity in clinical practice, it is time‐consuming and subject to inter‐rater variability, highlighting the need for standardized educational tools for raters [6]. The newly developed AI software demonstrated AUCs > 0.700 across all severity levels (≥ 1, ≥ 2, and 3). These results indicate that the software can reliably discriminate EASI severity levels, performing comparably to board‐certified dermatologists. Accordingly, the CNN developed in this study has the potential to support specialist‐level training and enhance educational effectiveness in EASI scoring.

The accuracy for detecting signs with an EASI component score of ≥ 2—the study's primary endpoint—showed moderate discriminative performance, with all AUCs calculated for individual EASI signs exceeding 0.800. For signs with a severity score of 3, the AUC, sensitivity, and specificity were even higher than those observed at lower levels. This trend may reflect the greater visibility and distinctiveness of more severe lesions in clinical images, making them easier for the AI to detect and classify. In contrast, accuracy was lower when evaluating the broader level of a severity score of ≥ 1. This is likely because this endpoint includes mild cases, which are more challenging for the AI to classify. This may be partly attributable to the characteristics of the dataset, which included images primarily from patients undergoing treatment, many of whom had relatively mild disease. Furthermore, because patients with a total EASI score of 0 were excluded from this study, the dataset did not include images of completely lesion‐free skin. This lack of a true “normal skin” baseline may have limited the model's ability to distinguish mild abnormalities from near‐normal skin. Incorporating a broader range of image data, including cases with a total EASI score of 0, could enhance the model's sensitivity to subtle clinical findings and improve performance for early‐stage disease.

In our AI‐based assessment of AD severity, the CNN achieved the highest AUC for erythema among the evaluated lesion features. This strong performance is likely attributable to the distinct color changes associated with erythema, which are readily captured in standard clinical photographs. Color‐based features typically provide a strong and easily learnable signal for CNNs, as supported by previous studies reporting high inter‐rater agreement for erythema and comparatively lower performance for texture‐based features, such as lichenification [12, 17]. Excoriation demonstrated the second‐highest AUC, suggesting that these lesions are also relatively straightforward for CNNs to detect. A plausible explanation is that excoriations, which appear as linear scratch marks, form edge‐like patterns that CNNs can readily identify. This observation aligns with findings in ophthalmology, where CNN‐based models have been shown to effectively recognize curvilinear structures such as retinal blood vessels [18]. These results suggest that line‐like features are inherently well‐suited to computer vision tasks, and the strong model performance for excoriation likely reflects its ability to leverage these geometric patterns. In contrast, papulation/edema yielded the lowest AUC among the four signs for the primary endpoint (a severity score of ≥ 2) and for severe cases (a severity score of 3). These lesions are inherently raised and three‐dimensional, making them more challenging to evaluate in two‐dimensional images. Nevertheless, although papulation/edema was the lowest‐performing sign among the four assessed signs, the model still achieved moderate‐to‐high performance, suggesting that severity assessment based on standard two‐dimensional color photographs is feasible to a meaningful extent. This performance may reflect the model's ability to capture relevant shadow and structural cues. Future work may explore whether incorporating depth information available on some smartphones can further improve performance while maintaining portability and feasibility. Additional improvements may also be possible by expanding the diversity of the training dataset to enhance robustness across varying lighting conditions.

In terms of clinical relevance, using uncropped and unmasked images is essential for real‐world implementation. However, images captured under uncontrolled lighting conditions and various angles introduce substantial challenges for AI, such as variations in shadows, colors, and perspectives. These variations can lead to unstable feature extraction and reduced accuracy. This is precisely why previous studies using CNN methods each relied on expert input to mitigate these issues: Bang et al. [12] used cropped lesion patches, Pan et al. [13] used an automated region‐of‐interest detector trained on trial‐derived cropped images, and Attar et al. [14] relied on dermatologist‐delineated lesion masks throughout training. To overcome these challenges directly, our study implemented robust methodological strategies. We employed the ConvNeXt architecture, a modern CNN known for its high performance and structural efficiency. Furthermore, we applied extensive data augmentation techniques—including random affine transformations, flipping, blurs, histogram equalization, and optical/grid distortions—to train a model resilient to the variability inherent in real‐world images. A key strength of this study is its multicenter design, which, enabled by these techniques, allowed for the collection of a large and diverse dataset of smartphone images captured without cropping or masking. To the best of our knowledge, this is the first study to evaluate AI‐based EASI severity scoring across multiple institutions using real‐world images obtained using a smartphone. The accuracy of this AI software, validated against the consensus of a Central Assessment Committee of board‐certified dermatologists, provides a strong foundation for its utility. This approach not only enhances the generalizability of the findings but also demonstrates the feasibility of deploying AI tools for standardized and scalable assessment and training in dermatologic severity evaluation. The accuracy observed against assessments by a Central Assessment Committee composed of board‐certified dermatologists supports the potential use of this software as a standardized educational tool for the EASI sign assessment. The present system was developed for educational purposes and has not been validated or optimized for direct clinical use; therefore, potential clinical applications, such as telemedicine, would require rigorous validation in future studies.

This study has some limitations. First, severity classification levels were determined by maximizing the Youden Index for each level (≥ 1, ≥ 2, and 3). Although effective for binary classification tasks, this method may be less suitable for multiclass classification involving levels 1, 2, and 3. This could lead to lower precision and bias towards over‐ or underestimation of severity levels, with the magnitude dependent on class distribution. Future work should explore alternative thresholding strategies to optimize multiclass performance. Second, the Youden Index prioritizes sensitivity and specificity, which can lead to lower positive predictive values in imbalanced datasets, particularly for severity level 3. This may lead to an overestimation of severe cases (increased false positives), with the magnitude more pronounced for rarer categories. In clinical applications, it is also important to consider PPV when determining thresholds for detecting high‐severity signs. Third, the dataset was collected exclusively from medical institutions in Japan, with the majority of images from Japanese patients. This introduces a generalizability bias, suggesting the AI's accuracy might be reduced in non‐East Asian populations owing to variations in skin phototypes and lesion morphology. The exact magnitude of this reduction is uncertain, necessitating further investigation. Therefore, the model's generalizability to non‐East Asian populations remains uncertain and will require additional studies to confirm its applicability. Fourth, our model does not estimate affected body surface area and therefore cannot provide a total EASI score. Although segmentation‐based area estimation may be possible with rigorous annotations, full‐body imaging may be impractical because of the associated time burden and patient consent/privacy considerations. Another potential approach would be to photograph representative, easily accessible body areas, such as the face, back, and extremities, and use these images to estimate the full EASI score. Fifth, AD was diagnosed by board‐certified dermatologists at each participating site as part of routine clinical practice, and no single standardized set of diagnostic criteria was mandated by the study protocol, which may have introduced inter‐site variability. Although this approach reflects diverse real‐world clinical settings, future work should explore employing standardized diagnostic criteria to validate the model.

In summary, in this multicenter study, we developed an AI software capable of evaluating EASI severity using images that required no cropping, masking, or controlled imaging conditions. The software demonstrated AUCs > 0.700 for a severity score of ≥ 1 and AUCs > 0.800 for severity scores of ≥ 2 and 3, indicating clinically acceptable performance. With its low‐cost and scalable design, this AI tool may offer a practical solution for training healthcare professionals and promoting standardized, efficient education in EASI severity assessment. Further validation is necessary to confirm its generalizability across broader clinical environments and diverse patient populations.

## Funding

This work was supported by Pfizer Japan Inc.

## Ethics Statement

The study was conducted in accordance with the Declaration of Helsinki and international ethical guidelines, including those of the Council for International Organizations of Medical Sciences. It also adhered to the Ethical Guidelines for Medical and Biological Research Involving Human Subjects, along with relevant Japanese laws and regulations, including the Act on the Protection of Personal Information.

*Approval of the research protocol by an Institutional Reviewer Board*: The study protocol was approved by the Takahashi Clinic Ethical Committee.

*Registry and the Registration No. of the study/trial*: This study is registered with the University Hospital Medical Information Network (UMIN) Clinical Trials Registry (Registration No. UMIN000052645).

## Consent

All patients provided written informed consent prior to participation.

## Conflicts of Interest

K.Y., Y.I., M.H., and K.N. are employees of Pfizer Japan Inc. Y.I., M.H., and K.N. own stock in Pfizer Japan Inc. Y.P. is an employee of Pfizer Belgium. T.N. and K.O. are employees of M3 Inc. K.O. owns stock in M3 Inc., and holds a leadership role in its affiliate, M3 AI Inc. This study was funded by Pfizer Japan Inc. M3 Inc. received funding from Pfizer Japan Inc. for AI development, and Mebix Inc. received funding for providing support for the study, including study management.

## Supporting information


**Table S1:** Number of images analyzed by atopic dermatitis sign and severity level.
**Table S2:** Number of patients whose erythema severity is determined as ≥ 2 or < 2 by AI software and the Central Assessment Committee.
**Table S3:** Number of patients whose papulation/edema severity is determined as ≥ 2 or < 2 by AI software and the Central Assessment Committee.
**Table S4:** Number of patients whose excoriation severity is determined as ≥ 2 or < 2 by AI software and the Central Assessment Committee.
**Table S5:** Number of patients whose lichenification severity is determined as ≥ 2 or < 2 by AI software and the Central Assessment Committee.
**Table S6:** Diagnostic accuracy of AI software for each sign of atopic dermatitis with an EASI component score of 3.
**Table S7:** Diagnostic accuracy of AI software for each sign of atopic dermatitis with an EASI component score of ≥ 1.

## Data Availability

Research data are not shared.
