# Supplementary material for: Educational Artificial Intelligence Software to Support Assessment of Atopic Dermatitis Severity
Source: J Dermatol. 2026 Jul 30;53(11):1572–80. doi: 10.1111/1346-8138.70434 (PMC13737445; doi:10.1111/1346-8138.70434)
Supplement: Supplementary file 1 [file JDE-53-1572-s001.docx]

Supplementary Materials

**Supplementary Tables**

| Severity level | Erythema | Papulation/Edema | Excoriation | Lichenification |
| --- | --- | --- | --- | --- |
| 0.0 | 327 | 648 | 1,449 | 855 |
| 1.0 | 2,082 | 1,500 | 1,406 | 1,406 |
| 1.5 | 579 | 638 | 403 | 630 |
| 2.0 | 588 | 718 | 365 | 562 |
| 2.5 | 78 | 109 | 47 | 150 |
| 3.0 | 22 | 63 | 6 | 73 |

**Supplementary Table 1. Number of images analyzed by atopic dermatitis sign and severity level**

**Supplementary Table 2. Number of patients whose erythema severity is determined as ≥2 or <2 by AI software and the Central Assessment Committee**

|  |  | Severity score by the Central Assessment Committee | | | | | | Total |
| --- | --- | --- | --- | --- | --- | --- | --- | --- |
|  |  | 0 | 1 | 1.5 | 2 | 2.5 | 3 |  |
| Severity score by AI software | ≥2 | 17 | 400 | 287 | 468 | 74 | 21 | 1,267 |
|  | <2 | 310 | 1,682 | 292 | 120 | 4 | 1 | 2,409 |
| Total | | 327 | 2,082 | 579 | 588 | 78 | 22 | 3,676 |

“Total” indicates the sum of the number of images in each row and column.

Abbreviation: AI, artificial intelligence.

**Supplementary Table 3. Number of patients whose papulation/edema severity is determined as ≥2 or <2 by AI software and the Central Assessment Committee**

|  |  | Severity score by the Central Assessment Committee | | | | | | Total |
| --- | --- | --- | --- | --- | --- | --- | --- | --- |
|  |  | 0 | 1 | 1.5 | 2 | 2.5 | 3 |  |
| Severity score by AI software | ≥2 | 37 | 348 | 286 | 508 | 96 | 59 | 1,334 |
|  | <2 | 611 | 1,152 | 352 | 210 | 13 | 4 | 2,342 |
| Total | | 648 | 1,500 | 638 | 718 | 109 | 63 | 3,676 |

“Total” indicates the sum of the number of images in each row and column.

Abbreviation: AI, artificial intelligence.

|  |  | Severity score by the Central Assessment Committee | | | | | | Total |
| --- | --- | --- | --- | --- | --- | --- | --- | --- |
|  |  | 0 | 1 | 1.5 | 2 | 2.5 | 3 |  |
| Severity score by AI software | ≥2 | 205 | 607 | 246 | 313 | 46 | 6 | 1,423 |
|  | <2 | 1,244 | 799 | 157 | 52 | 1 | 0 | 2,253 |
| Total | | 1,449 | 1,406 | 403 | 365 | 47 | 6 | 3,676 |

**Supplementary Table 4. Number of patients whose excoriation severity is determined as ≥2 or <2 by AI software and the Central Assessment Committee**

“Total” indicates the sum of the number of images in each row and column.

Abbreviation: AI, artificial intelligence.

|  |  | Severity score by the Central Assessment Committee | | | | | | Total |
| --- | --- | --- | --- | --- | --- | --- | --- | --- |
|  |  | 0 | 1 | 1.5 | 2 | 2.5 | 3 |  |
| Severity score by AI software | ≥2 | 73 | 345 | 280 | 388 | 139 | 68 | 1,293 |
|  | <2 | 782 | 1,061 | 350 | 174 | 11 | 5 | 2,383 |
| Total | | 855 | 1,406 | 630 | 562 | 150 | 73 | 3,676 |

**Supplementary Table 5. Number of patients whose lichenification severity is determined as ≥2 or <2 by AI software and the Central Assessment Committee**

“Total” indicates the sum of the number of images in each row and column.

Abbreviation: AI, artificial intelligence.

**Supplementary Table 6. Diagnostic accuracy of AI software for each sign of atopic dermatitis with an EASI component score of 3**

|  | AUC | 95% CI | | Sensitivity | Specificity | PPV | NPV |
| --- | --- | --- | --- | --- | --- | --- | --- |
|  |  | Lower | Upper |  |  |  |  |
| Erythema | 0.951 | 0.914 | 0.989 | 0.955 | 0.906 | 0.058 | 1.000 |
| Papulation/Edema | 0.901 | 0.867 | 0.935 | 0.905 | 0.806 | 0.075 | 0.998 |
| Excoriation | 0.959 | 0.923 | 0.994 | 1.000 | 0.879 | 0.013 | 1.000 |
| Lichenification | 0.909 | 0.881 | 0.937 | 0.890 | 0.822 | 0.092 | 0.997 |

Abbreviations: AI, artificial intelligence; AUC, area under the receiver operating characteristic curve; CI, confidence interval; EASI, Eczema Area and Severity Index; NPV, negative predictive value; PPV, positive predictive value.

**Supplementary Table 7. Diagnostic accuracy of AI software for each sign of atopic dermatitis with an EASI component score of ≥1**

|  | AUC | 95% CI | | Sensitivity | Specificity | PPV | NPV |
| --- | --- | --- | --- | --- | --- | --- | --- |
|  |  | Lower | Upper |  |  |  |  |
| Erythema | 0.810 | 0.787 | 0.833 | 0.698 | 0.795 | 0.972 | 0.204 |
| Papulation/Edema | 0.832 | 0.816 | 0.849 | 0.713 | 0.803 | 0.944 | 0.375 |
| Excoriation | 0.780 | 0.765 | 0.795 | 0.702 | 0.730 | 0.800 | 0.615 |
| Lichenification | 0.778 | 0.761 | 0.795 | 0.692 | 0.718 | 0.890 | 0.414 |

Abbreviations: AI, artificial intelligence; AUC, area under the receiver operating characteristic curve; CI, confidence interval; EASI, Eczema Area and Severity Index; NPV, negative predictive value; PPV, positive predictive value.
